# Factors Predisposing to Development of Dumping Syndrome in Post Operative Bariatric Patients: Experience from a Tertiary Unit

**DOI:** 10.1007/s11695-026-08657-7

**Published:** 2026-04-14

**Authors:** Arkeliana Tase, Alan Askari, Mariam Abed, Debbie Mussendeki, Omer Al-Taan, Aruna Munasinghe, Farhan Rashid, Tanveer Adil, Periyathambi Jambulingam, Douglas Whitelaw

**Affiliations:** 1 Department of Surgery and Cancer, Imperial College London, London, UK; 2 Bedfordshire Bariatric Unit, Luton and Dunstable University Hospital NHS Foundation Trust, Luton, UK; 3 Bedfordshire Bariatric Unit, Luton and Dunstable University Hospital NHS Foundation Trust, Luton, UK; 4 Department of Surgery and Cancer, Imperial College London, London, UK

## Abstract

**Background:**

Dumping syndrome (DS) is a recognised complication after bariatric surgery but reported prevalence and associated risk factors vary widely. This study aimed to determine the prevalence of DS and to identify predisposing factors in post-operative bariatric patients.

**Methods:**

A prospective observational cohort study was conducted at a high-volume bariatric unit in the UK. Consecutive patients reviewed between January 2024, and June 2025 were assessed using the Sigstad questionnaire. Data collected included type of surgery, pre- and post-operative weight, body mass index (BMI), percentage total body weight loss (%TBWL), and presence of diabetes mellitus (DM). Statistical significance was set at *p* < 0.05.

**Results:**

A total of 358 patients were reviewed (median age 45 years, 88% women). Thirty-six patients (10%) scored ≥ 7, meeting criteria for DS. The majority (91.7%) developed symptoms within one year. Revisional surgery was associated with a significantly higher risk of DS (22% vs. 8.7%, *p* < 0.01). No association was found with pre-existing DM (19% vs. 27%, *p* = 0.327). Patients with DS had lower post-operative BMI (33.2 vs. 40.5 kg/m²) and greater %TBWL (17.9% vs. 12%), though differences in absolute weight and BMI did not reach significance. An additional 11 patients (3.4%) developed transient, diet-related symptoms that resolved spontaneously.

**Conclusions:**

DS was present in 10% of patients, with a further 3.4% experiencing transient symptoms. Revisional surgery emerged as an independent risk factor, whereas no relationship with diabetes was observed. Early multidisciplinary input is recommended for high-risk patients, and multicentre studies are needed to standardise diagnostic and reporting methods.

## Key Points

Dumping syndrome affected 10% of patients, with most cases arising in the first post-operative year.

Revisional surgery was significantly associated with increased risk of DS.

No correlation was found between DS and pre-existing diabetes.

Patients with DS demonstrated greater percentage total body weight loss (%TBWL).

## Introduction

Dumping syndrome (DS) was first described in 1913 by Hertz and remains a recognised complication following bariatric surgery. The underlying mechanism involves rapid gastric emptying of hyperosmolar food into the jejunum, triggering gastrointestinal and vasomotor symptoms [1–8]. Although reactive hypoglycaemia is a common feature, other mechanisms such as non-insulinoma pancreatogenous hypoglycaemia syndrome and insulinoma have also been described. Dilatation of the gastrojejunostomy after Roux-en-Y gastric bypass (RYGB) may further contribute [9–11].

DS can occur after any bariatric procedure, including sleeve gastrectomy (SG), although the preserved pylorus is thought to lower risk [4, 12]. Bariatric surgery also influences gut hormones and appetite regulation, indirectly contributing to DS development [13]. Symptoms may occur early (within one hour of eating) or late (1–2 h post-prandially). Early symptoms include abdominal pain, diarrhoea, nausea, and vasomotor features such as flushing and palpitations. Late symptoms reflect hypoglycaemia, including hunger, sweating, tremor, and syncope [2, 4, 7, 14, 15] resulting in a significant impairment in the quality of life for the patient [14].

Reported prevalence of DS varies widely (10–50%) partly due to inconsistent definitions and scoring systems [3, 15, 16]. Some studies suggest transient symptoms that resolve within 18–24 months, while others debate its role in weight loss [3, 17].

Diagnostic tools include the Sigstad score [18], Dumping Symptom Rating Scale [14, 19], and Dumping Severity Score [20, 21], with Sigstad being most widely used.

An earlier preliminary pilot study carried out by our unit on 64 patients demonstrated a DS rate of 25%, with higher risk in patients with pre-existing diabetes and those undergoing revisional surgery [22]. In this prospective study aimed to validate these findings in a larger cohort, assessing prevalence, timing, surgical and metabolic risk factors, and transient symptoms.

## Methods

### Study Design and Setting

This was a prospective observational cohort study conducted at a high-volume bariatric centre in the United Kingdom (Luton and Dunstable NHS Trust, Bedfordshire Bariatric Unit). The unit prospectively maintains a secure database of all bariatric procedures, elective and emergency, in compliance with General Data Protection Regulation (GDPR).

### Patient Follow-up and Data Collection

All consecutive post-operative bariatric patients reviewed in outpatient clinic between January 2024, and June 2025 were eligible for inclusion. All patients reviewed in the bariatric clinic in this time period were included regardless of the date of surgery. The median follow up period of this study was 1 year. All patients seen in the bariatric clinic were included. All patients were reviewed in clinic by a bariatric specialist nurse, dietician or surgeon. No patients were lost to follow up.

Pre-operative weight, body mass index (BMI), and the presence of diabetes mellitus were obtained from clinic records. At each follow-up, patients were systematically assessed for symptoms of dumping syndrome (DS). From the outset of this project, bariatric specialist nurses administered the validated Sigstad scoring questionnaire [18] at all outpatient reviews. Patients were routinely followed up both by bariatric specialist nurses (in person or by telephone) and by the surgical team.

The sigstad scoring system is a diagnostic tool designed to aid in identification of dumping syndrome following gastric surgery. It evaluates a number of symptoms related to dumping and provides an overall score with a score of at least 7 being considered diagnostic.

Demographic information, surgical procedure, and follow-up dates were recorded, including the first presentation of DS symptoms. The Sigstad questionnaire was formatted in Microsoft Excel to facilitate structured completion by clinical nurse specialists, with total scores subsequently calculated by the principal investigator. A score ≥ 7 was considered diagnostic of DS, while a score < 4 was deemed suggestive of an alternative diagnosis requiring further investigation. Prospectively collected data were entered and verified by three investigators (AT, AA, MA) and stored in a secure institutional database.

### Statistical Analysis

Data analysis was performed using SPSS Statistics (version 31, IBM Corp., Armonk, NY, USA). Continuous variables were compared using independent samples t-tests, while categorical variables were compared using Chi-square tests. Statistical significance was set at *p* < 0.05.

### Sources of Bias

This study relied on accurate patient self-reporting of symptoms, introducing the possibility of reporting and recall bias. To mitigate this and reduce these biases, symptoms were documented contemporaneously using the Sigstad questionnaire by trained specialist nurses.

### Ethics and Reporting Standards

The study was registered as a quality improvement project with the Luton and Dunstable NHS Trust Education Department. Reporting followed the STROBE (Strengthening the Reporting of Observational Studies in Epidemiology) checklist for observational studies.

## Results

### Patient Characteristics

Between January 2024 and June 2025, 358 patients were reviewed following bariatric surgery. The median age was 45 years (IQR 37–54), and 88% were women. Thirty-six patients (10%) met the diagnostic threshold for dumping syndrome (DS) with a Sigstad score ≥ 7, of whom 97% (*n* = 35) were women (Table 1).

**Table 1:** Patients diagnosed with DS on Sigstad scoring system

Sigstad score > 7	Overall	36	10%
	Men	1	3%
	Women	35	97%
	RYGB	21	58.4%
	SG	7	19.4%
	Revision surgery	8	22.2%

### Surgical Procedures and Revisional Surgery

Roux-en-Y gastric bypass (RYGB) was the most frequently performed operation (*n* = 240, 67.6%), followed by sleeve gastrectomy (SG) (*n* = 80, 22.3%) and conversion of SG to RYGB (*n* = 30, 8.38%). Less common procedures included conversion to duodenal switch (*n* = 1), revisional RYGB (*n* = 2), conversion of one-anastomosis gastric bypass to RYGB (*n* = 1), and conversion of gastric band to RYGB (*n* = 2). A total of 36 patients (10.1%) underwent revisional procedures. The prevalence of DS was significantly higher in this group, with 22.2% of DS patients compared with 8.7% of non-DS patients having undergone revision (*p* < 0.01). Table 2 shows the data collected in this study.

**Table 2:** Types of surgery and pre-existing diabetes

Index Operation	RYGB	240	67%
	SG	80	22.3%
	Conversion SG to RYGB	30	8.38%
	Conversion gastric band to RYGB	2	0.6%
	Revision of RYGB	2	0.56%
	RYGB to Duodenal Switch	1	0.32%
	Conversion of OAGB to RYGB	1	0.32%
Revisional Surgery	Overall	36	10.1%
	DS cohort	8	22.2%
	Non DS cohort	28	8.7%
Pre-existing diabetes	Overall	94	26%
	DS cohort	7	19%
	Non DS cohort	87	27%

*RYGB – Roux en Y Gastric Bypass, SG – Sleeve gastrectomy, OAGB – one anastomosis gastric bypass

The main indication for revisional surgery was severe reflux (*n* = 27), GJ perforation/ulcer (*n* = 2) followed by chronic leak (*n* = 2), DS (*n* = 2), weight regain (*n* = 2) and functional obstruction (*n* = 1).

Standard limb lengths of 60–70 cm biliopancreatic limb and 105–110 cm alimentary limb were carried out on both primary and revisional surgeries. Our protocols are standardised with little variation between surgeons. No protocol changes were applied during this study. During revisional surgery (SG to RYGB) the pouch was created around a size 38Fr calibration tube and resized if required. We revised the pouch on 6 ( 20%) of these cases.

### Metabolic Risk Factors

Diabetes mellitus was present in 26% of the overall cohort. No significant association was observed between diabetes and DS: 19% of patients in the DS cohort had diabetes compared with 27% in the non-DS cohort (*p* = 0.327). The median pre-operative weight was 126.5 kg (IQR 112.5–142.3). Patients who developed DS had lower median pre-operative weight (117.6 kg) and BMI (42 kg/m², IQR 30.5–38.5) compared with the non-DS cohort (128 kg, BMI 46.45 kg/m², IQR 36.1–45.8), though differences were not statistically significant (weight *p* = 0.405, BMI *p* = 0.324). Post-operatively, the overall mean weight was 109.3 kg (IQR 95.5–125.2). Patients with DS had lower post-operative weight (92.2 kg, IQR 79.1–107.4) and BMI (33.2 kg/m², IQR 30.5–38.5) compared with those without DS (112.15 kg, BMI 40.5 kg/m², IQR 36.1–45.8). While differences showed a clear trend, they did not reach statistical significance, likely due to variability in outcomes. The mean percentage total body weight loss (%TBWL) for the overall cohort was 12% (IQR 10–16). Patients with DS achieved significantly greater %TBWL than those without DS (17.9% vs. 12%).(Table 3).

**Table 3:** Patient demographics and outcome data

		*n*	%
Sex	Male	43	12%
	Female	315	88%
Age (median, IQR)	45 yrs [37-54yrs]		
Pre-op weight (median, IQR)/ kg	Overall	126.5 kg [IQR 112.5-142.3].	
	DS group	117.6 kg [IQR 79.1–107.4]	
	Non DS	128 kg [IQR 113.4- 143.4]	
Pre-op BMI (kg/m2)	Overall	46 [IQR 41.3–51.6]	
	DS group	42 Kg/m2 [IQR 30.5–38.5]	
	Non DS	46.45 kg/m2 [IQR 36.1–45.8]	
Post op weight (median, IQR)/ kg	Overall	109.3 kg [IQR 95.5-125.2]	
	DS group	92.2 kg [IQR 79.1-107.4]	
	Non DS	112.15 kg [IQR 97–126]	
Post-op BMI (kg/m2)	Overall	40 kg/m2 [IQR 35.2–45.1]	
	DS group	33.2 kg/m2 [IQR 30.5–38.5]	
	Non DS	40.5 [IQR 36.1–45.8]	
%TBWL	Overall	12%	
	DS cohort	17.9%	
	Non-DS cohort	12%	

* DS – dumping syndrome, %TBWL – percentage total body weight loss

### Timing and Symptom Profile

Most patients with DS (*n* = 33, 91.7%) developed symptoms within the first post-operative year. Three patients presented later, at 4, 5, and 6 years after surgery, potentially reflecting under-recognition or intermittent symptoms managed in the community. An additional 11 patients (3.4%) experienced transient DS-like symptoms, triggered by dietary indiscretion (e.g., fatty foods, sweet desserts, or rapid eating). These resolved spontaneously following dietary modification and were not classified as DS due to their non-persistent nature.

## Discussion

This study demonstrated a prevalence of dumping syndrome (DS) of 10% within our bariatric cohort, with an additional 3.4% of patients experiencing transient symptoms that resolved with simple dietary adjustments. These transient symptoms were typically triggered by fatty foods, sweet desserts, or rapid eating. Laurenius et al. also observed that fat ingestion could provoke DS-like symptoms in the absence of the glucose and insulin rise associated with carbohydrate intake [23], highlighting the importance of dietary counselling in symptom management.

Dietary behaviour is an established factor in DS. Alsulami et al. reported that larger meal size significantly increased risk (81.8% vs. 10.9%) and that drinking liquids with meals, compared with between meals, also increased incidence (40.8% vs. 26.8%) due to accelerated gastric emptying [16]. Such findings reinforce the role of dietetic input in both prevention and treatment.

Our data demonstrated a strong association between revisional surgery and DS (22.2% vs. 8.7%, *p* < 0.01), consistent with prior studies showing higher rates of late dumping after revision compared with primary procedures [14, 24]. As demand for revisional surgery increases, this finding has important clinical implications for pre-operative counselling and long-term follow-up. Early involvement of dietitians and multidisciplinary teams (MDTs) is likely to mitigate risk and optimise outcomes in this high-risk subgroup. Larger studies are required to further confirm this in a wider group of patients.

We did not identify a significant relationship between pre-existing diabetes mellitus and DS. While this finding aligns with some published series, larger multicentre cohorts with a higher proportion of diabetic patients will be required to address this question definitively.

Patients who developed DS in our cohort achieved greater percentage total body weight loss (%TBWL) (19.1% vs. 13.2%). This contrasts with other studies who found no relationship between DS and %TBWL after RYGB [3, 7, 25]. The association between DS and weight loss remains unclear, and we advocate for future studies to adopt %TBWL as a more accurate and standardised measure than raw weight or BMI, given the heterogeneity of bariatric populations.

The relationship between DS and primary procedures is complex. DS is generally considered more common after RYGB than after SG, due to preservation of the pylorus in the latter [4, 12]. In our series, 56% of DS patients had undergone RYGB, while 19% developed DS following SG. Rates of DS after SG vary widely in the literature: Poljo et al. reported 15.6% [26], whereas Sun et al. [27] observed early DS in 40.9% and identified an association with low LDL cholesterol levels. 29 Similarly, Tzovaras et al. reported DS in 29% of SG patients, with a further 16% showing suggestive symptoms [8]. Our lower rates may reflect the predominance of RYGB in our unit and the smaller number of SG procedures performed.

Our findings also reinforce that DS most often occurs early: 91.7% of cases presented within the first post-operative year, with a smaller proportion developing late symptoms. Early dumping was the predominant presentation, although nearly one-third of affected patients experienced both early and late symptoms. This is consistent with Papamargaritis et al., who reported early dumping in 40% of SG patients at six months, persisting in 33% at twelve months, with late dumping increasing over time [21]. Such findings suggest that structured and sustained post-operative follow-up is critical for early recognition and intervention.

Other factors influencing DS include technical aspects of surgery. A multicentre prospective study demonstrated that the use of a linear stapler for gastrojejunal anastomosis increased DS risk compared with hand-sewn techniques (17.9% vs. 2.7%) [28]. This highlights the multifactorial aetiology of DS, encompassing patient, surgical, and metabolic factors.

### Study Limitations

This was a single-centre study conducted over a defined period. The predominance of women in the cohort (88%) limited meaningful sex-specific analyses, as only one male patient developed DS. A larger study is required to carry out a sex-specific analysis in more detail. Finally, although data collection was prospective, longer follow-up and multicentre studies will be needed to validate our findings and explore unanswered questions, including the role of diabetes and detailed surgical technique. The study relied on patients reporting their symptoms correctly and in a timely manner which introduces recall and reporting bias. We tried to mitigate these biases by asking the questionnaire questions on every clinic review however we are aware that they cannot be fully excluded.

## Conclusion

In this prospective cohort, 10% of patients developed DS after bariatric surgery, with a further 3.4% experiencing transient symptoms that resolved spontaneously. Revisional surgery was strongly associated with DS, whereas no correlation was found with pre-existing diabetes. Patients with DS demonstrated greater %TBWL, although differences in absolute weight and BMI were not statistically significant.

Given the growing prevalence of revisional bariatric surgery, early risk mitigation through MDT involvement, patient counselling, and close follow-up is essential. We recommend the use of %TBWL as a standardised metric for weight outcomes in DS research. Larger multicentre studies with longer follow-up are warranted to refine risk stratification, clarify metabolic associations, and inform best practice in the management of DS.

## Data Availability

No datasets were generated or analysed during the current study.
